# Examination of orthodontic expenditures and trends in the United States from 1996 to 2016: disparities across demographics and insurance payers

**DOI:** 10.1186/s12903-021-01629-6

**Published:** 2021-05-17

**Authors:** Man Hung, Sharon Su, Eric S. Hon, Edgar Tilley, Alex Macdonald, Evelyn Lauren, Glen Roberson, Martin S. Lipsky

**Affiliations:** 1 Roseman University of Health Sciences College of Dental Medicine, 10894 S. River Front Parkway, South Jordan, UT 84095 USA; 2 University of Utah School of Medicine, Salt Lake City, UT USA; 3 University of Chicago The College, Chicago, IL USA; 4 University of Utah Department of Mathematics, Salt Lake City, UT USA; 5 Portland State University Institute on Aging, Portland, OR USA

**Keywords:** Orthodontic expenditure, MEPS, Insurance coverage, Dental, Oral health, Orthodontics

## Abstract

**Background:**

Orthodontics prevent and treat facial, dental, and occlusal anomalies. Untreated orthodontic problems can lead to significant dental public health issues, making it important to understand expenditures for orthodontic treatment. This study examined orthodontic expenditures and trends in the United States over 2 decades.

**Methods:**

This study used data collected by the Medical Expenditure Panel Survey to examine orthodontic expenditures in the United States from 1996 to 2016. Descriptive statistics for orthodontic expenditures were computed and graphed across various groups. Trends in orthodontic expenditures were adjusted to the 2016 United States dollar to account for inflation and deflation over time. Sampling weights were applied in estimating per capita and total expenditures to account for non-responses in population groups.

**Results:**

Total orthodontic expenditures in the United States almost doubled from $11.5 billion in 1996 to $19.9 billion in 2016 with the average orthodontic expenditure per person increasing from $42.69 in 1996 to $61.52 in 2016. Black individuals had the lowest per capita orthodontic visit expenditure at $30.35. Out-of-pocket expenses represented the highest total expenditure and although the amount of out-of-pocket expenses increased over the years, they decreased as a percentage of total expenditures. Public insurance increased the most over the study period but still accounted for the smallest percentage of expenditures. Over the course of the study, several annual decreases were interspersed with years of increased spending

**Conclusion:**

While government insurance expenditure increased over the study period, out of pocket expenditures remained the largest contributor. Annual decreases in expenditure associated with economic downturns and result from the reliance on out-of-pocket payments for orthodontic care. Differences in spending among groups suggest disparities in orthodontic care among the US population.

## Background

Orthodontics is the field in dentistry that diagnoses, prevents, and treats facial, dental, and occlusal anomalies. If orthodontic conditions are left untreated, these anomalies can lead to significant dental problems such as tooth decay and periodontal disease, thus highlighting the importance of treatment. Many studies demonstrate that occlusal anomalies can predispose individuals to localized periodontal problems in cases of traumatic overbites, crossbites, overjets that increase risk for trauma, and tooth positioning that can comprise periodontal support [1]. Periodontal disease is a major cause of tooth loss and is independently associated with several systemic chronic inflammatory diseases [2]. Orthodontic intervention corrects these occlusal anomalies and may optimize periodontal therapy outcomes by correctly positioning teeth to increase the thickness of surrounding bone and tissues and improving gingival recession [3, 4]. Additionally, malocclusion negatively affects an individual’s ability to masticate and break down food [5]. Malocclusions can worsen over time and warrant timely orthodontic intervention [5, 6]. In addition, orthodontic treatment improves esthetics, and as such the popularity of orthodontic cosmetic care is increasing [7]. Each year over 9 million individuals in the United States receive orthodontic treatment [8], making it the third largest treatment category in dentistry [9].

The field of orthodontics has seen a number of changes over recent years. Although 75% of orthodontic patients are under 18 years of age, the demographic composition of orthodontic patients is changing and the number of adults under treatment is increasing. In 2018, an estimated 1.61 million adults received treatment in the United States, up from 1.55 million in 2016 [8]. Another change in orthodontic practice is how patients enter care. In the past, the biggest referral source for orthodontic care was from general dentists [10]. However, self-referral and word of mouth are becoming more common and an increasing number of patients also try home care first and then self-refer [11].

Fees, payments, and insurance coverage for orthodontic care vary widely. Typically, expenditures are grouped into different categories based on payment source. Public insurance payments include government funded coverages such as Medicaid, Medicare, worker’s compensation and Veterans Affairs (VA) related insurance. Private insurances include employer plans, Tricare, and individually purchased coverages.

Insurance plans vary in their covered services for orthodontic treatment. Medicaid covers only a handicapping malocclusion due to birth defects, accidents, disease or abnormal growth patterns, or conditions that that affect nutrition. Children’s Health Insurance Program (CHIP) provides dental coverage which includes procedures deemed medically necessary to prevent disease and promote oral health, to restore oral structures to health and function, and to treat emergency conditions [12]. Both CHIP and Medicaid leave the interpretation of “medically necessity” for orthodontic treatment up to the provider and each state [13]. Medicare typically does not cover orthodontic services with limited exceptions such as to treat conditions resulting from disease or injury. The VA offers dental coverage based on a veteran’s benefit level, but only covers dental services that are necessary for medical and oral health and usually does not cover orthodontic procedures [14].

Private insurance coverage for orthodontic procedures also varies and generally more extensive dental coverage incurs greater cost. For example, Delta Dental, a private dental insurance company, features three different plans. The most costly or premium benefit plan, lists orthodontic services at 50% coverage. The basic plan does not cover orthodontic procedures while the individual/family plan features a co-pay of about $2600 to $2800 for orthodontic services [15]. Privately insured individuals may purchase additional dental coverage and these individuals are more likely to visit the dentist and have higher expenditures [16].

Older reports indicate that most patients seeking orthodontic care were primarily uninsured and/or from a higher income population [17]. A 2010–2012 study found that 56% of the care for children was paid out of pocket [18], while children with public insurance only represented 9.4% of orthodontist visits [9]. While some dental insurance plans offer full or partial orthodontic coverage for care deemed “medically necessary,” the lack of standardization for determining qualified cases creates disparities among case approvals. The Affordable Care Act (ACA) mandated medically necessary orthodontia but then failed to define “medically necessary,” instead leaving the definition up to individual states [13]. Data also suggest disparities in care related to insurance status and the ability to pay [14]. For example, children with public assistance and minority children received the fewest orthodontic procedures [9], and many children needing orthodontic care are either underinsured or uninsured [19]. As much as 15% of the US population have orthodontic problems severe enough to affect function suggesting a gap between need and delivered care [20]. Among children, 17.2% demonstrated a definite need for orthodontic treatment and about one-third would likely benefit from care [21].

In terms of orthodontist supply, between 1995 and 2006, the number of orthodontists increased by 1315 with a 13.3% increase in orthodontic private practices [22]. Reflecting this increase in supply of orthodontists, the orthodontist to child (ages 5–17) ratio increased nationally from 16.9 to 17.7 per 100,000 children. Despite the national increase, this ratio varied across states ranging from 9.2 in Mississippi to 36.0 in the District of Columbia. Ten states experience decreased ratios, highlighting major differences in orthodontist distribution across the country despite national increases in practicing orthodontists, suggesting unmet need.

The combination of unmet need and cost as a potential barrier to care makes it important to understand orthodontic expenditures. Though previous studies examined orthodontic expenditures, there has been recent changes to orthodontic practice related to self-care. More adults are now seeking care, as well as having new approaches to fees and payments, and new guidelines have made it important to update earlier research and to explore cost trends over time. In addition, earlier studies were limited because their research design focused on limited samples, settings, or narrow time frames. Using national samples that were representative of the United States population, the purpose of this descriptive study was to examine orthodontic expenditures, insurance coverages, and to explore trends in expenditures in the United States over the past 2 decades. This study augments the existing literature by updating expenditures, by assessing the impact of changes affecting orthodontic practice on expenditures, and identifying if disparities exist in orthodontic utilization based on race/ethnicity, poverty level, and insurance status.

## Materials and methods

This descriptive study examined orthodontic care expenditures from 1996 to 2016 using the Medical Expenditure Panel Survey (MEPS) Household Component as the source to obtain longitudinal data. MEPS represents the United States civilian, non-institutionalized population and utilizes annual questionnaires to collect data on individual household members and families in regards to demographics, health status, socioeconomic aspects, and access to care. MEPS is sponsored by the Agency for Healthcare Research and Quality and represents a complete data source on the health and dental expenditures by individuals and families in the nation. These annual questionnaires are designed to help provide more transparency about the nation’s fluctuating health care system. The detailed information gathered from these questionnaires was self-reported. Study participants take part in several rounds of interviewing where they report on changes in their health status, income, employment, use of services, payment, and eligibility for public and private insurance coverages. Since households may have difficulty reporting third-party payments, the MEPS supplements household reports of such payments with data obtained through a follow-back survey of providers [23]. More detailed information about MEPS and its validity and reliability can be found at https://www.meps.ahrq.gov/. Since the data were de-identified and available to the public, this study does not require review from the Institutional Review Board according to US federal regulations (45 CFR 46, category 4).

Data processing began with merging all MEPS data from the 1996 to 2016. Demographic characteristics were examined for all respondents from 1996 to 2016. Statistics for orthodontic expenditures were calculated and graphed across various groups such as age, marital status, race, gender, income and insurance coverage, along with orthodontic expenditures covered expenses by different insurance payers. For the purposes of this study, private insurance was defined as employer plans, and Tricare is the health care program for US uniformed service members, retirees, and their families around the world. Public health insurance plans in the US consists of federally funded government insurance plans for low-income individuals or families such as Medicaid, Medicare (for the elderly), and other individuals that qualify for special subsidies. Uninsured individuals were those without public or private insurances and who paid for fees out of pocket.

Both per capita and total expenditures were calculated. Total expenditure was computed by adding up all of the expenditures from 1996 to 2016. Total expenditure divided by the population sample size is the per capita expenditure. Dollar amounts were adjusted to the year 2016 to account for inflation and deflation over the study period and to allow for comparability across all years. The adjustment used the inflation and deflation values published by the United States Bureau of Labor Statistics. In order for the data sample to be representative of the United States’ population, sampling weights were applied when estimating the expenditures. Using this approach was necessary in order to account for the non-responses in certain population groups. All statistics were computed using the R software version 4.0.2.

## Results

### Study sample

Table 1 summarizes the demographic characteristics of the study population. Of 690,298 participants from 1996 to 2016, the mean age was 34.5 years old (standard deviation = 22.4) with 25.6% of the study population aged under 18 years. Among the study sample population 52.2% were female and 37.3% married. The mean income was $26,070 (standard deviation = $30,556). When excluding individuals who reported no expenditures, 56.2% of the orthodontic patients were under 18 years old.

**Table 1:** Demographic characteristics (N = 690,298)

Variable	Entire sample	Individuals reporting no expenditure	Individuals reporting expenditure > 0
Age in year, mean ± SD (range)	34.5 ± 22.4 (0–90)	34.7 ± 22.4 (0–90)	20.0 ± 14.4 (0–88)
Person’s total income in $, mean ± SD (range)	26,070 ± 30,556 (− 275,219–731,653)	26,226 ± 30,565 (− 275,219–731,653)	15,360 ± 27,911 (− 26,527, 437,861)
Sex, n (%)			
Male	329,689 (47.8%)	324,443 (47.9%)	5246 (40.9%)
Female	360,609 (52.2%)	353,043 (52.1%)	7566 (59.1%)
Marital status, n (%)			
Married	257,494 (37.3%)	255,906 (37.8%)	1588 (12.4%)
Widowed	33,302 (4.8%)	33,171 (4.9%)	131 (1.0%)
Divorced	53,923 (7.8%)	53,583 (7.9%)	340 (2.7%)
Separated	13,404 (1.9%)	13,333 (2.0%)	71 (0.6%)
Never married	155,357 (22.5%)	151,883 (22.4%)	3474 (27.1%)
Under 18 years old—N/A	176,283 (25.6%)	169,077 (25.0%)	7206 (56.2%)

Negative income is possible because MEPS allows reporting of negative income

### Trends in orthodontic expenditures

**Fig. 1:**
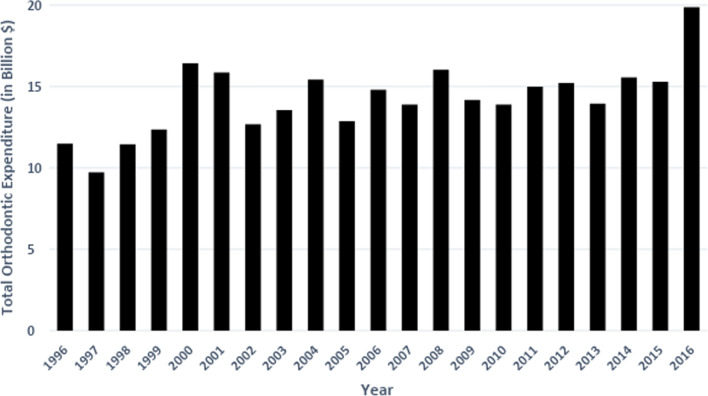
US total orthodontic expenditures from 1996 to 2016

Between 1996 and 2016, there was an overall increase in total orthodontic expenditures in the United States, with expenditures almost doubling (73% increase) from $11.5 billion in 1996 to $19.9 billion in 2016, when adjusted for inflation (Table 2/Fig. 1). The average orthodontic expenditure per person increased from $42.69 in 1996 to $61.52 in 2016, representing a 40% increase (Table 2). Over the course of the study period, a decrease in total orthodontic expenditures and average orthodontic expenditure per person occurred in the periods of 1996–1997, 2000–2002, 2004–2005, 2006–2007, 2009–2010, and 2012–2013, with the greatest decrease from $15.9 billion to $12.7 billion (30% decrease) occurring from 2001 to 2002 (Table 2). All other years exhibited an increase in total orthodontic expenditures, with the greatest increases seen from 1999 to 2000 (32% increase) and from 2015 to 2016 (30% increase) (see Table 2). Despite fluctuations, over longer intervals total orthodontic expenditures showed a gradual progressive increase during the study period (Fig. 1).

**Table 2:** Total orthodontic expenditures and average orthodontic expenditures in the United States from 1996 to 2016 (All amounts adjusted to 2016 dollars)

Year	Total orthodontic expenditures	Average orthodontic expenditure per person	Average orthodontic expenditure per person (expenditure > 0)
1996	11,482,174,261.36	42.69	1989.52
1997	9,721,472,173.56	35.83	1867.45
1998	11,450,974,171.39	41.86	2082.99
1999	12,357,602,269.96	44.70	2381.74
2000	16,432,428,941.06	59.03	2788.55
2001	15,866,652,679.33	55.83	2620.79
2002	12,672,969,530.81	43.97	2145.59
2003	13,547,791,416.87	46.62	2146.49
2004	15,419,535,095.51	52.54	2370.55
2005	12,871,054,410.62	43.45	2256.19
2006	14,804,110,223.06	49.47	2365.03
2007	13,888,433,413.10	46.09	2267.95
2008	16,036,707,284.41	52.68	2634.37
2009	14,174,342,571.67	46.23	2407.88
2010	13,896,912,642.50	45.04	2653.56
2011	14,983,588,076.46	48.16	2458.07
2012	15,208,427,538.90	48.51	2534.85
2013	13,954,668,311.86	44.20	1988.41
2014	15,562,667,213.90	48.88	2280.81
2015	15,298,532,515.92	47.59	2151.51
2016	19,879,895,500.85	61.52	2760.87

### Expenditures by insurance type

**Fig. 2:**
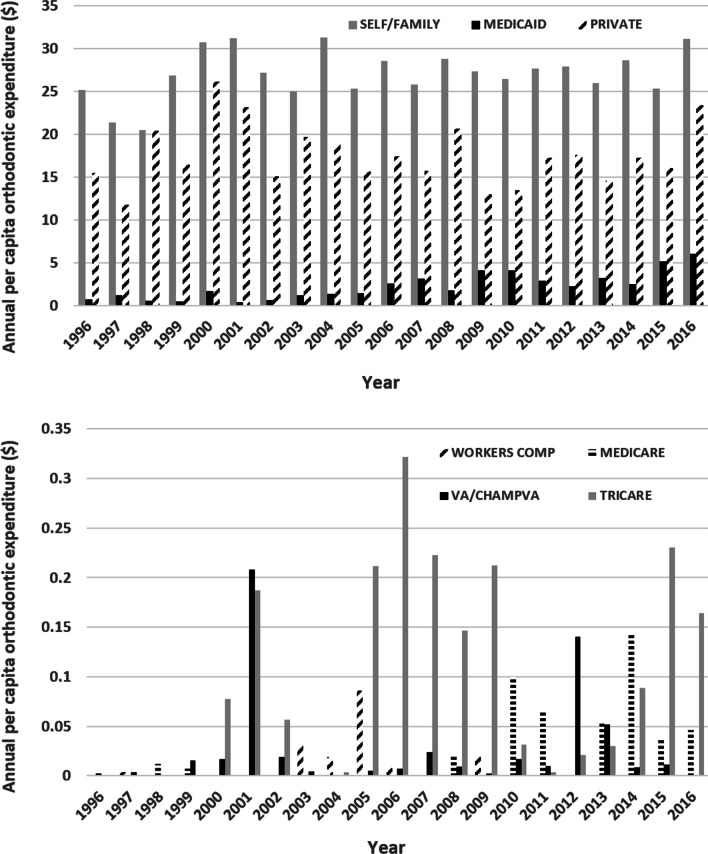
US per capita orthodontic expenditures by insurance coverage

Figure 2 depicts per capita of different insurance coverages used for orthodontic expenditures. Over the past 2 decades, per capita Medicaid expenditure increased substantially from $0.65 in 1996 to $5.98 in 2016, out-of-pocket per capital expenditure went from $25.15 to $31.16, while per capita Medicare expenditure went from $0.003 to $0.05. Out-of-pocket expenditures were the most common type of orthodontic payment throughout and although total out-of-pocket expenditures increased by $3.3 billion, they decreased as a percentage of total expenditures by 8% from 59% in 1996 to 51% in 2016. Private insurance expenditure fluctuated throughout the years. Prior to 2010, the average annual Medicare expenditure was $912,861 and average annual Medicaid expenditure was $436,806,257 in orthodontics. From 2010 to 2016, the average annual Medicare orthodontic expenditure was $20,201,930 and average annual orthodontic Medicaid expenditure was $1,178,598,306.

### Expenditures by demographic groups

**Fig. 3:**
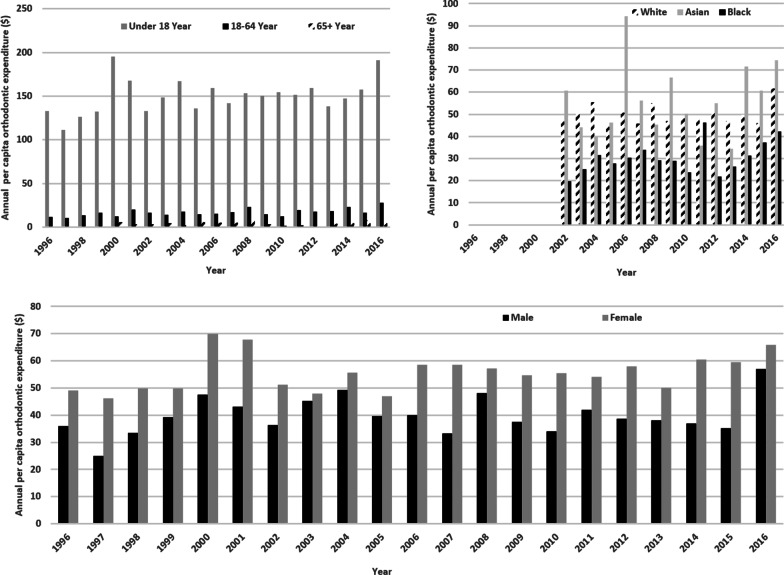
US per capita orthodontic expenditures by demographic groups

**Fig. 4:**
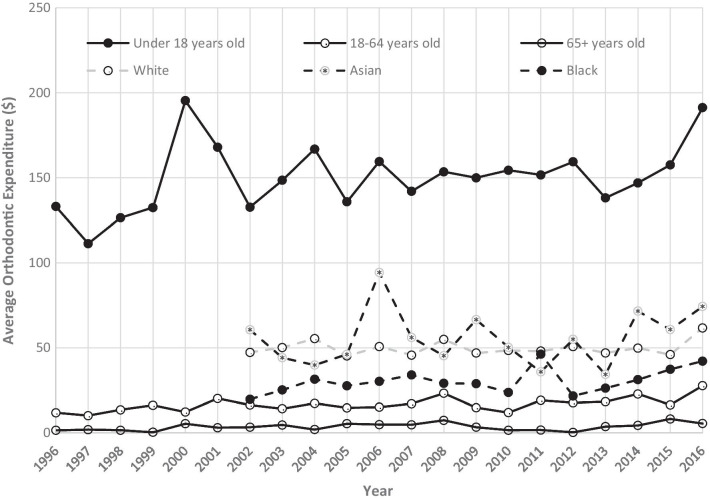
Average per capita orthodontic expenditures by age and racial groups (all cases)

**Fig. 5:**
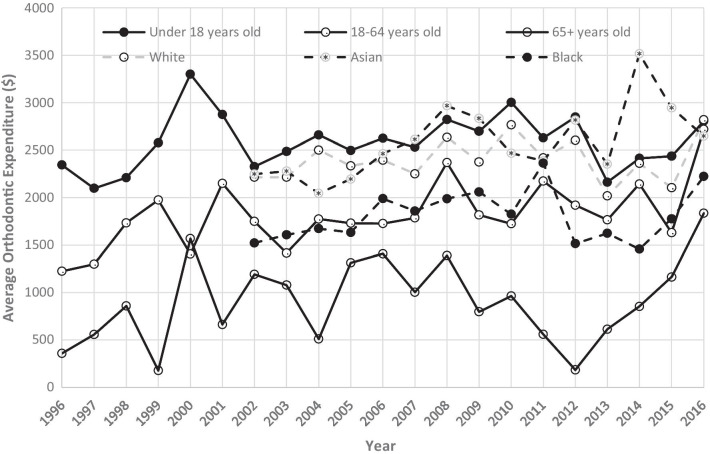
Average per capita orthodontic expenditures by age and racial groups (cases with expenditure > 0)

Figure 3 is a representation of the per capita of orthodontic expenditures across different subgroups from the year 1996 to 2016. Those under age 18 years displayed the highest per capita orthodontic visit expenditures, while adults over age 65 years exhibited the lowest spending. For marital status, the subgroup “never married” spent more per capita during most of the study period than the subgroups of “separated”, “widowed”, “married”, and “divorced”. However, in the year 2016, there was a spike in per capita orthodontic visit expenditures by “separated” individuals. This particular spike was approximately 10 times the amount of the previous year. Asian, Caucasian and Black all exhibited variation from year to year. Over the study period, the average orthodontic expenditure per person increased from $42.69 in 1996 to $61.52 in 2016 with Black individuals having the lowest per capita orthodontic visit expenditure at $30.35. The same pattern remained true that the Black individuals had the lowest average orthodontic expenditure whether our analyses included cases with expenditure > 0 or all cases (Figs. 4, 5).

## Discussion

Using a nationally representative database this study found that there was an overall increase of 8.4 billion dollars in orthodontic expenditures over the last 2 decades. Total orthodontic expenditures in the United States almost doubled from $11.5 billion in 1996 to $19.9 billion in 2016. In contrast, over the same period dental care expenditures increased by 27% and per capita healthcare expenditures increased by 60% [24]. One explanation for this increase may be related to an increase in the number of orthodontists. However, in contrast to the 8.4% increase in expenditure, the number of orthodontists per 100,000 population over the study period only increased by 0.45%. This study also found that orthodontic expenditures in public spending accounts such as Medicare and Medicaid substantially increased after the enactment of Affordable Care Act in 2010. Documenting existing expenditure, trends and out-of-pocket expenses provides useful information to policy makers and insurers about the cost of expanding coverage. Costs have important health implications since more than 20% of children would benefit from orthodontic treatment [25]. Treatment contributes to the public health by identifying and managing malocclusions that can compromise nutrition, lead to gum disease and bone erosion and contribute to breathing disorders such as sleep apnea and improving quality of life [26].

Another key finding was the variation seen in expenditures over the study period with several year-to-year decreases interspersed with years of increased spending. Although both total dental and medical expenditures show some fluctuations over this same time period, they vary to a lesser degree [24]. This implies that, because of its dependence on out-of-pocket payments, orthodontic care is more of a luxury, and more sensitive to social and economic conditions such as the housing crisis, the tech bubble burst, the global recession, and events like the 9/11 terrorist attacks which correlated with a 30% drop in orthodontic visits. It is possible that cosmetic care is more sensitive to economic changes, but regardless the fluctuations highlight the importance of safety net funding and expanding public funding for orthodontic care especially during times of crisis to avoid financially vulnerable patients with medical necessity going untreated. It also suggests that a standard definition of medical necessity will help focus resources to provide care to those with most in need of treatment during an unfavorable economic climate.

Total expenditures from all payment sources increased throughout the 20-year time frame. While out-of-pocket payments represented the largest expenditure, they decreased as percent of total expenditure. Public insurance expenditures, which predominantly cover children, increased at a steeper rate than other forms of payment, suggesting that policy makers and legislators are beginning to recognize the importance of orthodontic care. However, the persistent disparity between federal coverage and out-of-pocket expenditures raises questions on whether insurance policies appropriately match community need for treatment. Since higher income individuals and those with private insurance were more likely to receive care, augmenting federal insurance coverage for orthodontic care appears to be one solution to narrowing the gap between care and need.

Study results found that individuals under 18 years old exhibited the greatest orthodontic expenditures per capita, a finding similar to other studies. This is consistent with the American Association of Orthodontics recommendation to have an orthodontic evaluation by age 7 to detect problems and to begin treatment between ages 9 and 16 in order to optimize treatment and prevent later complications [27]. Expenditures were greater for females than males throughout the entire study period, a finding also consistent with previous reports. One possible explanation is that females are more likely to seek cosmetic care than their male counterparts [28]. A surprising finding was that Blacks exhibited the greatest year-to-year fluctuation in expenditure and there was a sharp peak for Asians orthodontic expenditure in 2016. This suggests that Blacks and Asians might be the most vulnerable population to economic downturns, with the greatest need for interventions and supportive programs during crisis periods. Further study is needed to confirm these findings and to explore possible reasons and solutions.

Like all studies, this study has several limitations. MEPS samples the civilian, noninstitutionalized population, so it does not include the 5% of those individuals institutionalized in the US. Nonetheless, MEPS remains the most complete medical expenditure database in the United States and our results should be generalizable to 95% of the US population. Another issue is that since MEPS uses a computer interface for interviews, household reporting may not be recorded accurately due to a lack of technical knowledge in using computers from some households [29]. An additional limitation is that MEPS reports on expenditure data but does not include indirect costs such as time off or travel costs related to doctor appointments [30]. MEPS data also do not distinguish between orthodontic care that is medically necessary versus cosmetic care and do not identify to what extent newer direct-to-patient aligner treatments and aligner treatments offered by dentists contribute to expenses. Finally, self-reported responses may reflect personal bias; however, the follow-back survey of providers helps to reduce potential bias.

## Conclusions

From 1996 to 2016, total orthodontic expenditures in the United States almost doubled. Expenditures fluctuated with several year-to-year decreases interspersed with years of increased spending, suggesting that orthodontic expenditures are sensitive to the economic environment and may be related to the high proportion of self-pay patients. Differences in spending among groups suggest disparities in orthodontic care among the US population.

## Data Availability

The datasets generated and/or analyzed during the current study are available in the Medical Expenditure Panel Survey (MEPS) Household Component repository, https://www.meps.ahrq.gov/.
